# Diversification of spatiotemporal expression and copy number variation of the echinoid *hbox12/pmar1/micro1* multigene family

**DOI:** 10.1371/journal.pone.0174404

**Published:** 2017-03-28

**Authors:** Vincenzo Cavalieri, Fabiana Geraci, Giovanni Spinelli

**Affiliations:** 1 Department of Biological, Chemical and Pharmaceutical Sciences and Technologies (STEBICEF), University of Palermo, Viale delle Scienze Edificio 16, Palermo, Italy; 2 Advanced Technologies Network Center (ATeN), University of Palermo, Viale delle Scienze Edificio 18, Palermo, Italy; Laboratoire de Biologie du Développement de Villefranche-sur-Mer, FRANCE

## Abstract

Changes occurring during evolution in the *cis*-regulatory landscapes of individual members of multigene families might impart diversification in their spatiotemporal expression and function. The archetypal member of the echinoid *hbox12/pmar1/micro1* family is *hbox12-a*, a homeobox-containing gene expressed exclusively by dorsal blastomeres, where it governs the dorsal/ventral gene regulatory network during embryogenesis of the sea urchin *Paracentrotus lividus*. Here we describe the inventory of the *hbox12/pmar1/micro1* genes in *P*. *lividus*, highlighting that gene copy number variation occurs across individual sea urchins of the same species. We show that the various *hbox12/pmar1/micro1* genes group into three subfamilies according to their spatiotemporal expression, which ranges from broad transcription throughout development to transient expression in either the animal hemisphere or micromeres of the early embryo. Interestingly, the promoter regions of those genes showing comparable expression patterns are highly similar, while differing from those of the other subfamilies. Strikingly, phylogenetic analysis suggests that the *hbox12/pmar1/micro1* genes are species-specific, exhibiting extensive divergence in their noncoding, but not in their coding, sequences across three distinct sea urchin species. In spite of this, two micromere-specific genes of *P*. *lividus* possess a TCF/LEF-binding motif in a similar position, and their transcription relies on Wnt/β-catenin signaling, similar to the *pmar1* and *micro1* genes, which in other sea urchin species are involved in micromere specification. Altogether, our findings suggest that the *hbox12/pmar1/micro1* gene family evolved rather rapidly, generating paralogs whose *cis*-regulatory sequences diverged following multiple rounds of duplication from a common ancestor.

## Introduction

The last two decades of research in the field of molecular embryology have provided a detailed mechanistic explanation of how fates of different cell types are encoded in the genome and sculpted through the sequential progression of transcriptional states of defined regulatory genes [1–4]. By contrast, much less is known about the driving forces underlying dynamic rewiring of gene regulatory network circuitries during evolution [5–9]. In this regard, it is commonly accepted that gene duplication provides a major source of both evolutionary novelty and species diversification. In fact, in all three domains of life a substantial fraction of genes underwent a series of duplications that originated multicopy gene families [10,11], and among these is the echinoid *hbox12/pmar1/micro1* family. *Hbox12* was originally identified in the Mediterranean *Paracentrotus lividus* species as a cDNA coding for a paired-like class homeodomain-containing factor [12]. Whole mount in situ hybridization and *cis*-regulatory analysis highlighted that the archetypal *hbox12* gene, namely *hbox12-a*, is expressed transiently during the very early cleavage stages exclusively by presumptive dorsal blastomeres [12–15]. We also showed that *hbox12-a* encodes a key transcription repressor functioning at the top of the symmetry-breaking sequence of events within the dorsal-ventral gene regulatory network. In particular, by transient inactivation of p38-MAP kinase activity during very early cleavage, Hbox12-a defines the future dorsal side of the embryo, allowing the expression of the TGF-β superfamily member Nodal on the opposite side [14–16]. Afterwards, Nodal-dependent signaling imposes the dorsal-ventral polarity in the developing embryo [17–20].

To date, proteins that show high sequence similarity to the Hbox12-a regulator are encoded by the *pmar1/micro1* genes identified in *Strongylocentrotus purpuratus* [21], *Hemicentritus pulcherrimus* [22], *Anthocidaris crassispina* [23], and *Lytechinus variegatus* [24]. Similarly to *hbox12-a*, multiple copies of *pmar1*/*micro1* genes are clustered in the respective sea urchin genomes [13,23,25]. However, the Pmar1 factor is produced solely by micromeres, where it drives their specification by inhibiting transcription of the ubiquitous repressor HesC, which otherwise negatively regulates the repertoire of micromere specification genes [26–28]. The fact that the Hbox12-a and Pmar1/Micro1 regulators display high sequence similarity across species but serve different functions poses the question of whether diversification of their *cis*-regulatory sequences has arisen during evolution by duplication of a common ancestor.

In the current paper, we address this question by describing the inventory of the *hbox12/pmar1/micro1* genes present in *P*. *lividus*, and highlighting that gene copy number variation occurs across the genome of distinct individual sea urchins of the same species. We also show that members of this gene family exhibit extensive divergence in their noncoding, but not in their coding, sequences among three different urchin species, as well as substantial differences of spatiotemporal expression during embryogenesis of *P*. *lividus*. While some members coherently recapitulate the animal-restricted expression pattern formerly described for the *hbox12-a* gene [12,13], others are either transcribed broadly throughout development or transiently expressed only in micromeres of the fourth cleavage embryo, mimicking the expression pattern reported for *pmar1*/*micro1* in other species. Furthermore, a predicted TCF/LEF-binding motif exists in a similar position, compared to *pmar1* genes of *S*. *purpuratus*, near the transcription start site of the micromere-specific genes of *P*. *lividus*, and the expression of these genes specifically relies on the nuclerization of β-catenin in vegetal blastomeres. We propose that the echinoid *hbox12/pmar1/micro1* family includes distinct paralogs that most likely evolved independently following multiple rounds of duplication from a common ancestor.

## Materials and methods

### Quantitative PCR analysis

For gene copy number estimation, genomic DNA was purified from sperm of seven individual urchins, and then used as template in SYBR Green-based qPCR reactions for *hbox12/pmar1/micro1* genes (see below). As known single copy controls we used the *otp* and *cmpl* genes [29,30]. The number of *hbox12*, *otp* and *cmpl* amplicons was determined using standard curves with different dosages of plasmid DNA containing the mentioned amplicon sequences, and gene ratios were eventually calculated. The primers for the assay are listed in S1 Table.

For gene expression analysis, reverse-transcription and qPCR were performed essentially as described [15,31,32]. Briefly, total RNA from batches of dissected or injected embryos grown at the desired stage was extracted using the Power SYBR Green Cells-to-CT kit (Ambion) and reverse transcribed following the manufacturer’s recommendations. The resulting cDNA sample was further diluted and the equivalent amount corresponding to one embryo was used as template for qPCR analysis, with the oligonucleotide primers indicated in S1 Table. qPCR experiments were performed from at least three distinct batches and all reactions were run in triplicate on a StepOnePlus Real-Time PCR System (Thermo Fisher Scientific) using SYBR Green detection chemistry. ROX was used as a measure of background fluorescence and, at the end of the amplification reactions, a melting-curve analysis was run to confirm the homogeneity of all amplicons. Calculations from qPCR raw data were performed using the StepOne software version 2.3 (Thermo Fisher Scientific), with the comparative Ct method. Primer efficiencies were found to exceed 1.85. In each experiment, a no-template control was included for each primer set. A *cytochrome oxidase* or the *mbf1* mRNA, which are known to be expressed at a constant level during development, was used to normalize all data, in order to account for fluctuations among different preparations. The number of transcripts per embryo at the very early blastula stage was estimated assuming a reference standard number of 1000 copies/embryo of the *z12* mRNA [33,34].

### Embryo dissection and microinjection of synthetic mRNA

Embryo dissection was carried out as described [14,35]. Briefly, unperturbed *P*. *lividus* embryos nearing the end of the fourth cleavage were transferred into a modified Kiehart chamber in Ca^2+^-free sea water and manipulated with fine glass needles under a Leica M165FC stereomicroscope equipped with micromanipulators (Narishige). Groups of three embryos at one time were manipulated by removing micromeres immediately following their formation from the overlying macromeres. After surgery, the isolated micromeres and their complementary micromere-less embryoids, as well as control intact 16-cell stage embryos, were promptly processed for qPCR analysis.

Microinjection was conducted as described [36,37]. *ΔLvG-Cad* [38], *dnTCF* [39], and *strim* out-of-frame [35] constructs were linearized and used as template to synthesize capped mRNAs using the mMessage mMachine kit (Ambion). Approximately 1–2 pl of the purified RNAs were injected in 30% glycerol at the following concentrations: *ΔLvG-cad* 0.1 pg/pl, *dnTCF* 0.4 pg/pl, and *strim* out-of-frame 0.5 pg/pl.

For each dissection or injection experiment, roughly one hundred embryos were processed for qPCR analysis, and each experiment was repeated twice with different batches of eggs.

## Results

### Inventory of the hbox12/pmar1/micro1 homeobox genes residing in the P. lividus genome

**Fig 1:**
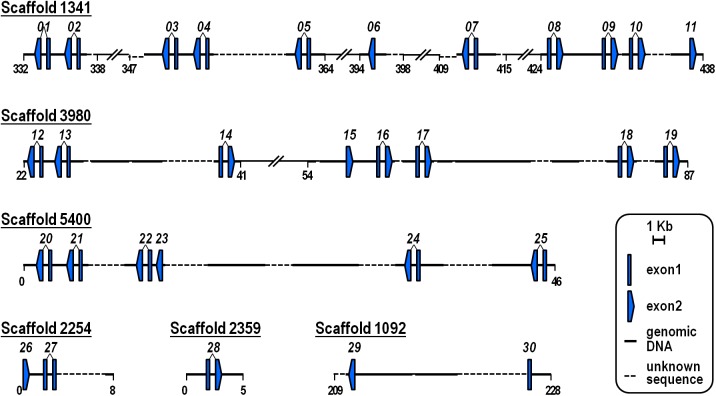
Genomic organization of the *hbox12/pmar1/micro1* family members of the sea urchin *P*. *lividus*. The exon-intron structure, orientation, and location for each gene are shown. Scaffold number identifiers and coordinates are based on v4.0 of the *P*. *lividus* genome assembly (http://octopus.obs-vlfr.fr/blast/oursin/blast_oursin.php). The *hbox12-27* gene displays a normal genomic organization, but the 3’ portion of the exon 2 is missing due to lack of genomic sequence. Unrelated genes are omitted for simplicity.

As a first step toward identification of the complete set of *hbox12/pmar1/micro1* genes of the Mediterranean sea urchin *P*. *lividus*, an in-silico analysis was carried out using the most recent assembly of the genome (v4.0, 01/2016; http://octopus.obs-vlfr.fr/blast/oursin/blast_oursin.php). A BLASTn search retrieved a total of thirty hits distributed in six distinct genomic scaffolds of different size (Fig 1). Neither *hbox12-a* nor *-b*, the two originally identified genes [13], were found among these hits, probably because in some cases the gene anatomy was partially obscured by the incomplete nature of the available genome draft. Nevertheless, Genscan analysis revealed that each of the thirty genes share an identical structure, which consists of two exons split by a single intron (Fig 1), mirroring the genomic organization formerly described for the prototypical *hbox12-a* and -*b* genes [13]. The only exception is the *hbox12-29* gene, for which the first exon has not been identified (Fig 1). The newly identified gene copies were often oriented divergently from one another, being transcribed outwardly in the opposite direction irrespective of their genomic location (Fig 1). Interestingly, some of the mentioned copies were arranged in contiguous bigenic clusters with a head-to-tail orientation (Fig 1), a peculiar feature that we observed previously in the genomic λ-clone containing the *hbox12-a* and *-b* genes [13]. The fact that all of the *hbox12/pmar1/micro1* family members share the same exon-intron structure, and that chimeric copies were not detected, indicates that gene units have presumably duplicated as intact copies. Importantly, the amino acid sequences predicted for the thirty genes showed extensive similarity to each other and to the Hbox12-a regulator, especially in the homeodomain (S1 Fig), with minor differences likely due to allelic polymorphisms.

The various *hbox12/pmar1/micro1* copies were flanked by unrelated genes that were apparently distinguished among scaffolds, indicating that the mentioned scaffolds were not overlapping, and that therefore the gene copy number obtained by the in silico analysis was not overestimated. Nevertheless, this possibility cannot be excluded because in the provisional genome draft technical complications hindered the separation of the two haplotypes mixed during preparation of the sequencing library (indeed, the current assembly size of 1.4 Gb is well above the expected 0.8 Gb for the haploid genome).

In a recent paper [40], the partial or complete protein sequences of five *hbox12/pmar1/micro1* family members were inferred from one contig of *P*. *lividus* genomic sequence. We found that two of these proteins, termed by the authors Hbox12-9 and -2, were 100% identical to those we deduced for the *hbox12-01* and *-20* genes, respectively (S2 Fig). However, in contrast to Hbox12-9 and -2, these genes map into distinct scaffolds (Fig 1), suggesting that they would lie in different genomic regions. Analogously, pairwise alignments showed that the proteins predicted for the *hbox12-02*, -*04* and -*27* genes displayed the highest similarity to the three remaining sequences, although various differences were detected (S2 Fig). Although these discrepancies could be at least in part due to allelic polymorphisms, in the absence of genomic sequence for the contig described in [40], we cannot rule out the possibility that we were looking at further distinct copies of the same gene family, which probably escaped from the sequencing and/or assembly of the *P*. *lividus* genomic reads.

**Fig 2:**
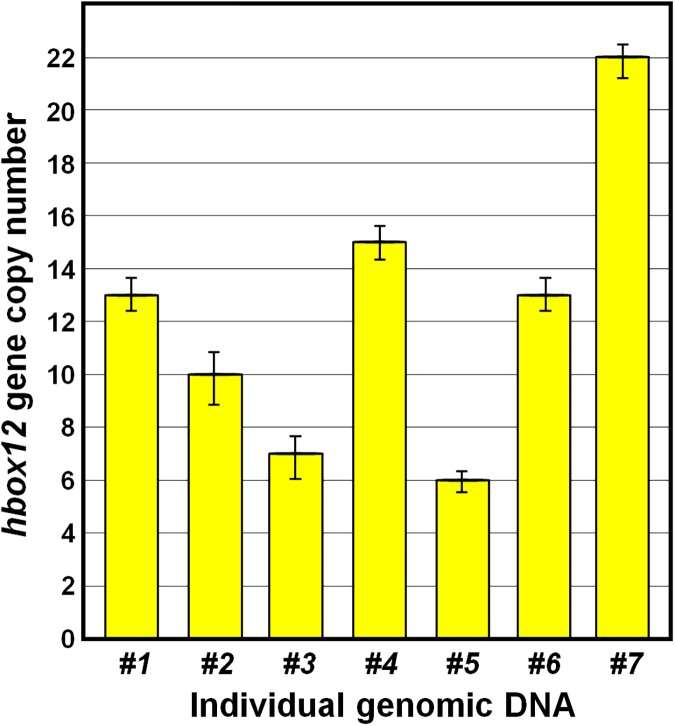
*Hbox12/pmar1/micro1* gene copy number determination in the genome of seven distinct *P*. *lividus* individuals. The histogram derived from qPCR data represent the number of *hbox12* copies normalized to the single copy gene controls *otp* and *cmpl*. Error bars are standard errors for the qPCR replicates.

Hence, to measure the *hbox12/pmar1/micro1* gene dosage in *P*. *lividus* more precisely, we performed qPCR using a universal primer pair that completely matched the sequence of all the known family members (S1 Table), and specific primers for the *otp* and *cmpl* genes [29,30], used as single copy controls. As a template for this assay, we utilized the genomes derived from sperm of seven randomly selected individuals and, much to our surprise, we found that the number of *hbox12/pmar1/micro1* genes varied noticeably among individuals, ranging from 6 to 22 copies (Fig 2).

Taken together, our findings not only confirm that several copies of these genes exist in the genome of *P*. *lividus*, but also suggest that the *hbox12/pmar1/micro1* family expanded via multiple episodes of segmental duplication.

### Heterogeneity of spatiotemporal expression profiles within the hbox12/pmar1/micro1 gene family

**Fig 3:**
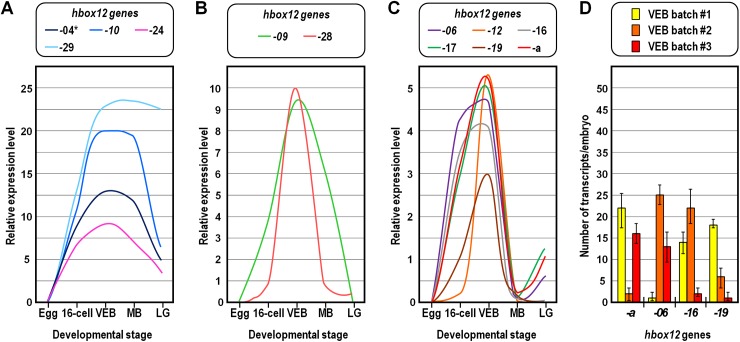
Temporal expression of the *hbox12/pmar1/micro1* gene family members in the *P*. *lividus* embryo. (A-C) Individual representation of each expression profile presented as the mean relative to global *hbox12/pmar1/micro1* transcript abundance measured with the universal primer pair at the indicated developmental stages. Expression profiles with standard errors of the mean between replicates are shown individually in S3 Fig. The developmental stages are as follows: Egg, unfertilized egg; 16-cell, fourth cleavage embryo; VEB, very early blastula; MB, mesenchyme blastula; LG, late gastrula. The asterisk indicates that the *hbox12-04* amplicon was obtained together with the *hbox12-21* and *-25* amplicons by co-amplification with the same primer pair. (D) The absolute number of transcripts per embryo given at the very early blastula stage derives from independent qPCR experiments using distinct cDNA batches. Further detail for the qPCR procedure is given in Materials and Methods. The error bars are standard errors for qPCR replicates. The oligonucleotide primer pairs used for the qPCR reactions and amplicon lengths are indicated in S1 Table.

The transcription level of about half of the *hbox12/pmar1/micro1* family members was assessed by qPCR analysis from unfertilized eggs and embryos at distinct developmental time points, using gene-specific primer pairs (S1 Table). The results of this survey revealed that transcripts derived from the examined genes are not maternally stored in the unfertilized egg (Fig 3 and S3 Fig), and that three zygotic expression groups are distinguishable on the basis of the abundance and temporal accumulation of transcripts during embryogenesis (Fig 3 and S3 Fig). The *hbox12*-*04*, -*10*, *-24*, and -*29* genes, altogether falling in a first group, were found to be transcribed at significantly higher levels throughout development (Figs 3A and S3). In the case of *hbox12*-*04*, however, the result could reflect the sum of separate transcriptional activities, as we noticed that the potential cDNAs derived from *hbox12*-*21* and -*25* genes are co-amplified using the *hbox12*-*04* primer pair. The *hbox12-09* and -*28* genes were both expressed at reduced levels when compared to the former group, and their transcripts were not detected at the late gastrula stage (Fig 3B and S3 Fig). A third group included *hbox12-a*, -*06*, -*12*, -*16*, -*17*, and -*19*, the genes displaying the lowest detectable expression levels among those investigated (Fig 3C and S3 Fig). It is also interesting to note that the mRNA abundance of most of the genes belonging to the third group varied significantly across the different cDNA batches used, and there was no apparent reciprocal correlation in the transcriptional status of these genes within a given cDNA sample (Fig 3D). Moreover, transcripts of the *hbox12*-*07*, -*08*, and -*14* genes were not detected at all in the cDNA batches we examined (not shown), suggesting that they were either absent or accumulated at an extent that cannot be determined under our experimental conditions. Altogether, these results indicate remarkable heterogeneity in gene expression occurring among both the *hbox12/pmar1/micro1* family members and the distinct population of embryos from which cDNA templates were derived.

**Fig 4:**
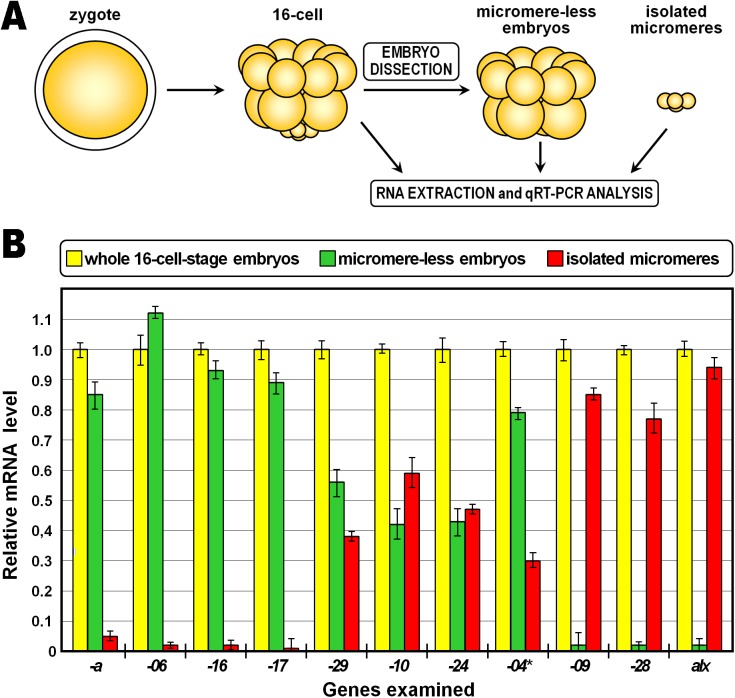
Spatial distribution of the *hbox12/pmar1/micro1* gene family transcripts in fourth cleavage embryos. (A) Fertilized eggs were grown until the 16-cell stage, followed by embryo dissection. Isolated micromeres and the complementary micromere-less embryos, as well as control undissected embryos at the same stage, were immediately processed for qPCR analysis. (B) qPCR measurements of *hbox12/pmar1/micro1* family members and *alx* transcript abundance in embryo fractions shown as a percentage of the *hbox12* and *alx* mRNA levels in control undissected embryos at the fourth cleavage. The error bars are standard errors for the qPCR replicates. Oligonucleotide primer pairs used for qPCR reactions and amplicon lengths are indicated in S1 Table.

As mentioned, the expression profile of the *P*. *lividus hbox12-a* gene differs from those of the *pmar1* and *micro1* genes described in *S*. *purpuratus* and *H*. *pulcherrimus*, respectively [12,13,21,22]. To distinguish the *hbox12-a-like* from the *pmar1/micro1-like* genes in *P*. *lividus*, we assessed the spatial distribution in the developing embryo of the transcripts derived from the various *hbox12/pmar1/micro1* family members. Unfortunately, collecting of probes that unambiguously recognize the mRNA of each copy in whole mount in situ hybridization assays was hampered by the very extensive identity of both coding sequences and flanking UTRs within the *hbox12/pmar1/micro1* family. To overcome this drawback, we combined a dissection approach to qPCR analysis, focusing on the 16-cell stage embryo, when the discordance in the spatial expression patterns of *hbox12-a* and *pmar1/micro1* genes is apparent [12,21,22]. Unperturbed *P*. *lividus* embryos were grown until the fourth cleavage, followed by micromere removal. Isolated micromeres and the complementary micromere-less embryos, as well as control undissected embryos at the same stage, were immediately processed for qPCR analysis (Fig 4A). As expected, the micromere-specific marker *alx1* [41], used as a control, was expressed in isolated micromeres at a level nearly equal to that of the whole embryos, and there was no evidence of *alx1* expression in micromere-less embryos (Fig 4B). By contrast, at the time of micromere removal the *hbox12*-*a* gene was selectively transcribed in micromere-less embryos, in perfect accordance with previous observations [13–15]. Other members of the family, such as *hbox12*-*06*, -*16*, and -*17*, grouped together coherently reiterating the spatial expression profile established for *hbox12-a* (Fig 4B). The remaining *hbox12/pmar1/micro1* copies were subdivided into additional groups according to their divergent spatial expression. In particular, mRNAs produced by *hbox12*-*10*, -*24*, and -*29* genes were conjointly detected in isolated micromeres and the micromere-less counterpart, in both cases at a similar reduced level compared to the sibling control intact embryos (Fig 4B), suggesting that probably the mentioned genes were either ubiquitously expressed or broadly transcribed along the animal-vegetal axis of unperturbed 16-cell stage embryos. Alternately, the transcript population derived from the *hbox12*-*04* gene was found in both the dissected embryonic fractions, although with preferential accumulation in micromere-less embryos (Fig 4B). In the absence of a specific primer pair (*hbox12*-*04*, -*21* and -*25* were co-amplified, as mentioned), this result should be considered as the sum of potentially separate transcriptional profiles. Finally, *hbox12*-*09* and -*28* transcripts were found to be specifically restricted to isolated micromeres (Fig 4B).

Collectively, these results highlight the complexity of spatiotemporal expression profiles achieved by the *hbox12/pmar1/micro1* family in the developing *P*. *lividus* embryo, leading us to further focus on those members showing the *hbox12-a-like* and *pmar1/micro1-like* expression patterns. In particular, we performed qPCR with specific primer pairs to determine the presence/absence of each of these subfamily members in the genome of the seven sea urchin individuals described in the previous section. We found distinct composition of the mentioned subfamilies in the genome of the examined individuals (Table 1), as expected from the high copy number variation of the *hbox12/pmar1/micro1* family. In light of this, it could be reasoned that genes that did not generate amplicons in the expression analyses were probably missing, rather than silenced, in those batches. However, it should be emphasized that a negative qPCR result for a given *hbox12/pmar1/micro1* gene does not necessarily mean that the gene is missing in the genome. Moreover, polymorphisms occurring in the sequences recognized by the primers could have hampered detection by our current qPCR assay.

**Table 1:** Composition of the *hbox12-a-like* and *pmar1/micro1-like* gene subfamilies in seven *P*. *lividus* individuals.

Gene sub-family	Gene name	Individual genomic DNA						
		#1	#2	#3	#4	#5	#6	#7
*hbox12-a-like*	*hbox12-a*	-	+	-	-	-	-	+
	*hbox12-06*	-	-	+	-	-	-	+
	*hbox12-12*	-	-	+	-	-	-	+
	*hbox12-16*	+	+	-	+	+	+	-
	*hbox12-17*	+	+	+	+	+	+	+
	*hbox12-19*	+	-	-	+	-	+	-
*pmar1/micro1-like*	*hbox12-09*	-	+	-	+	-	-	+
	*hbox12-28*	+	+	+	+	+	+	+

Whatever the explanation, the presence of *hbox12-17* and *-28*, respectively belonging to the *hbox12-a-like* and *pmar1/micro1-like* subfamilies, was determined in all the examined genomes (Table 1), suggesting that these two genes could constitute, possibly together with additional unidentified members, the core gene set of the *hbox12/pmar1/micro1* family.

### Relationships between hbox12/pmar1/micro1 genes across sea urchin species

**Fig 5:**
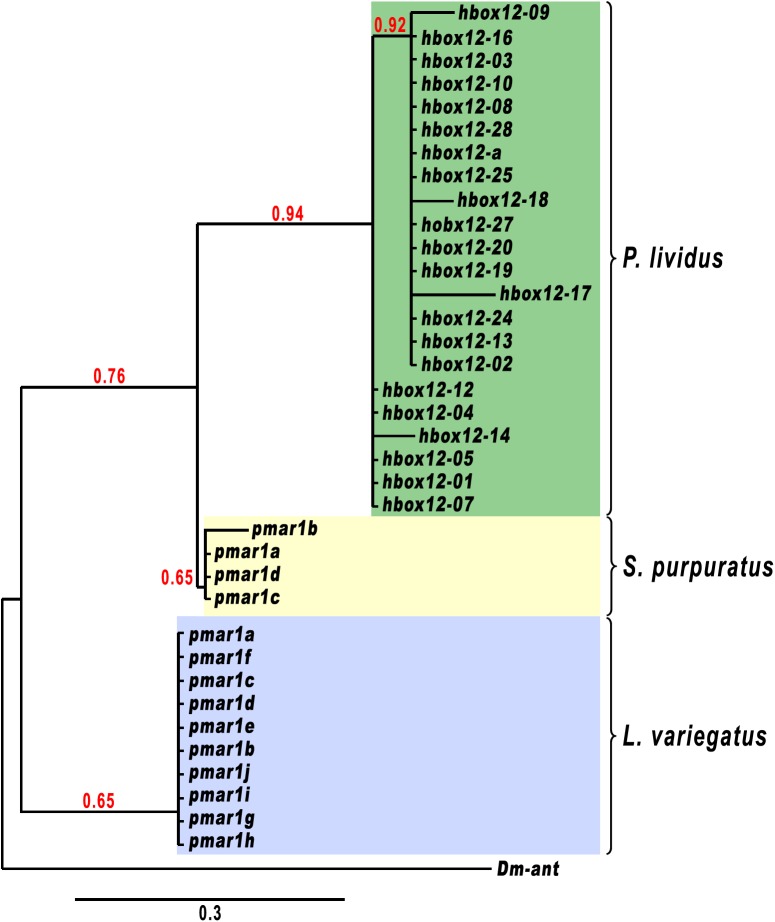
Phylogenetic relationship among the *hbox12/pmar1/micro1* genes of *P*. *lividus*, *S*. *purpuratus* and *L*. *variegatus*. After alignment of sequences with MUSCLE v3.8.31, ambiguous regions (i.e. containing gaps and/or poorly aligned) were removed with Gblocks v0.91b. A rooted Maximum Likelihood phylogenetic tree was reconstructed using the PhyML v3.1/3.0 aLRT, and graphically represented using TreeDyn v198.3. The *ant* gene (AY060407.1) of *Drosophila melanogaster* was identified by a BLAST search with *hbox12-a* against the NCBI databases as one of the homeobox-containing genes with higher sequence similarity within the homeobox, and it was therefore used as an outgroup. Numbers above nodes record percent bootstrap values, and branches with support value smaller than 50% are collapsed. *pmar1* genes from *L*. *variegatus* and *S*. *purpuratus* were retrieved by Genscan analysis of the following scaffold items: AC131562.1 (*Lvpmar1a-j*), AC168388.2 (*Sppmar1a-b*), AC179748.1 (*Sppmar1c*), and AC149920.2 (*Sppmar1d*).

To explore how the *hbox12/pmar1/micro1* genes evolved, we first used the Phylogeny.fr platform [42] to build a phylogenetic tree with a set of complete gene sequences (5’-flanking+exons+intron) available for the *P*. *lividus*, *S*. *purpuratus*, and *L*. *variegatus* species. Notably, in this analysis the various genes formed three distinct species-specific clades supported by reliable bootstrap values (Fig 5), suggesting that these genes diverged after the split among the mentioned sea urchin species.

**Fig 6:**
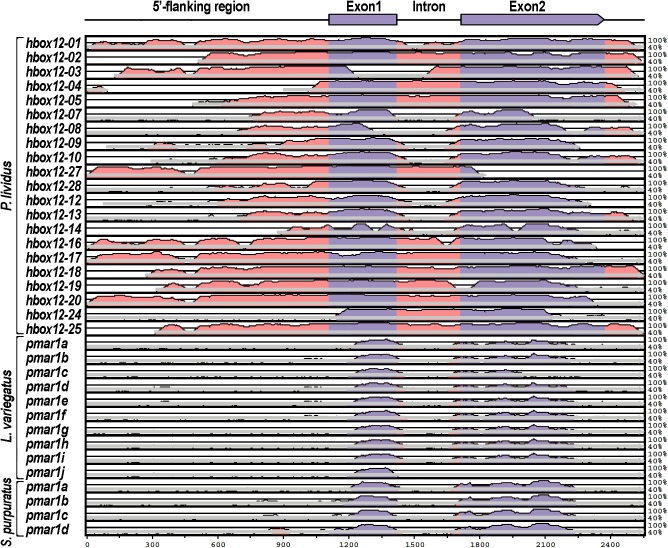
Comparison of *hbox12/pmar1/micro1* loci across sea urchin species. Structural annotation of the *hbox12/pmar1/micro1* locus is shown in the drawing on top. The genomic sequences used in Fig 5 were compared using the mVISTA software package [46,47] to determine evolutionary conserved regions among the three sea urchin species indicated, using *hbox12-a* as the reference sequence. Each graph show a pairwise alignment with the extent of sequence identity plotted on the Y-axis against the indicated sequence. The grey arrow below each graph shows the extent of the sequence used, while the filled portions indicate conservation (>70% over 100 bp) of either exons (labeled in blue) or noncoding sequences (pink). Note that significant sequence similarity across all species is found exclusively in the protein coding regions.

Consistently with this finding, a phylogenetic footprinting analysis using the mVISTA software package to compare the genomic sequences of the *hbox12/pmar1/micro1* family members of *P*. *lividus* with those of *S*. *purpuratus* and *L*. *variegatus*, failed to detect significant evolutionary conservation in their *cis*-regulatory apparatuses over the entire gene units, at any setting of the algorithm (Fig 6 and S4 Fig). As additional reassuring evidence that we were looking at unrelated loci, no syntenic association was detected between the genes surrounding the *hbox12/pmar1/micro1* genomic regions of the three sea urchin species (not shown).

Furthermore, from this analysis we appraised that the *hbox12/pmar1/micro1* family members of *P*. *lividus* could be subdivided into distinct categories on the basis of conservation of their promoter sequences (Fig 6), that generally correlated with the diversification of their expression profiles. For instance, the *hbox12*-*a*, *-16*, *-17*, and *-19* genes yielded fairly equivalent spatiotemporal expression and consistently exhibited pervasive conservation of their promoter sequences, differing in terms of both expression profile and promoter composition from the group formed by *hbox12*-*09* and -*28* genes (Figs 3, 4 and 6). The *hbox12-12* gene, which also belongs to the *hbox12-a-like* expression group, is an exception to this trend, displaying poor sequence conservation limited to the 5’-most portion of the promoter (Fig 6).

**Fig 7:**
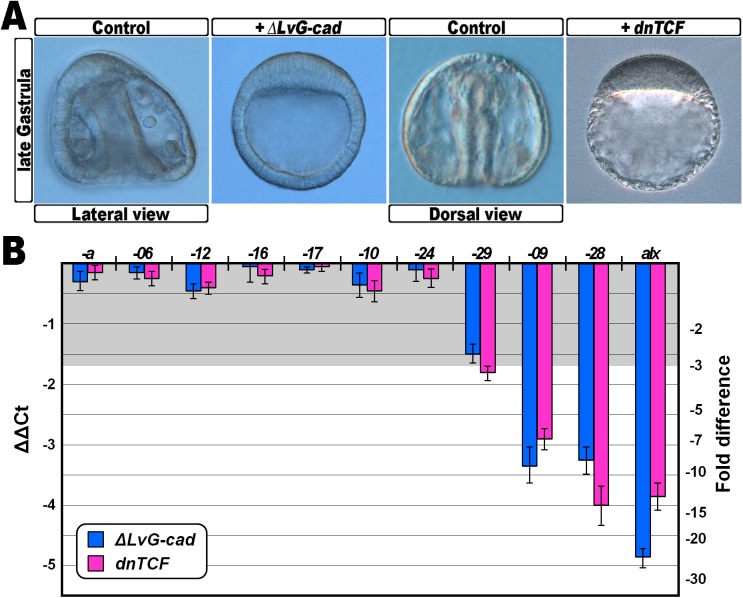
Inhibition of the wnt/β-catenin signalling pathway and effect of *hbox12/pmar1/micro1* family gene expression. (A) Representative examples of control gastrulae and embryos at the same stage injected with either *ΔLvG-cad* or *dnTCF* synthetic transcripts. (B) Changes in gene expression level of *hbox12/pmar1/micro1* family members assessed by qPCR in *ΔLvG-cad*- and *dnTCF*-injected embryos. Data are indicated as normalized ΔCt (ΔΔCt, left ordinate), and as the corresponding fold difference in transcript abundance (right ordinate), with respect to control embryos, at the same stage of development, derived from zygotes injected with the *strim1* out-of-frame transcript. The gray region represents ΔΔCt values corresponding to non-significant variation (less than 3-fold difference). Error bars are standard errors for the qPCR replicates. Oligonucleotide primer pairs used for qPCR reactions and amplicon lengths are indicated in S1 Table.

Similarly, the regulatory regions of *hbox12-09* and -*28* apparently diverged from those of the *pmar1/micro1* genes of other sea urchin species (S4 Fig), but maintained the capability to drive a similar spatiotemporal expression profile restricted to micromeres at the fourth cleavage stage. As a fundamental input triggering the expression of the genes required for the specification of the micromere-lineage is represented by the nuclearization of maternal β-catenin in vegetal blastomeres [23,26,39,41,43], we looked at the promoter sequences of both the *pmar1/micro1* and *hbox12-09*/-*28* genes for TCF/LEF-binding consensus sites. Interestingly, multiple canonical sequence motifs (TCAAAG) were predicted (S5 Fig), and one of them occupied a rather similar position with respect to the transcription start site of the mentioned *hbox12/pmar1/micro1* family members from *S*. *purpuratus* and *P*. *lividus* (S5 Fig), suggesting that these genes could be similarly regulated by the Wnt/β-catenin pathway. To assess this possibility, we used two different perturbation approaches. First, we disrupted this signal by overexpression of *ΔLvG-cad*, a synthetic mRNA encoding the transmembrane and intracellular domains of the cell adhesion molecule LvG-cadherin [38]. ΔLvG-cad binds and traps β-catenin in the cytoplasm, leading to depletion of the signalling pool of β-catenin [44,45]. *Strim out-of-frame*-injected controls [35] and *ΔLvG-cad*-injected embryos at the 60/120-cell stage were collected and processed for qPCR. An extra aliquot of *ΔLvG-cad*-injected embryos set aside and observed later in development, showed a typically animalized phenotype, whereas unperturbed embryos normally gastrulated (Fig 7A). As a control for this assay we used *alx1*, a known micromere-specific target of β-catenin [41], and as expected the mRNA abundance of *alx1* decreased abruptly following overexpression of ΔLvG-cad (Fig 7B). Although to a lower extent compared to *alx1*, a reduction in the transcriptional activity was also detected for the micromere-specific genes *hbox12*-*09*, -*28*, and for the broadly expressed gene *hbox12-29* (Fig 7B), suggesting that their maximal expression in the unperturbed embryo normally relies either directly or indirectly on the positive input given by nuclear β-catenin. This conclusion was also supported by equivalent results obtained following overexpression of dnTCF (Fig 7), a dominant negative form of the sea urchin TCF lacking the β-catenin binding domain [39]. Importantly, in both the perturbation assays we did not notice any significant reduction in the transcript amounts of the vast majority of the *P*. *lividus hbox12/pmar1/micro1* family members (Fig 7B), confirming that their expression occurs independently of the β-catenin nuclear internalization at the vegetal pole.

Taken together, these results would suggest that the *P*. *lividus hbox12*-*09*/-*28* and the *pmar1/micro1* genes of other species are regulated in a similar way by β-catenin dependent signalling.

## Discussion

### Evolutionary expansion and expression of the hbox12/pmar1/micro1 family members

Comparative genomic studies revealed that duplication of a gene encoding a transcription factor represents an effective strategy for a transcription factor to explore new functions during evolution, without significantly decreasing the fitness of an organism [48,49]. Although one of the duplicates could be loss by pseudogenization [50], different models have been proposed to describe the functional outcomes of duplicate genes [50–54]. In the subfunctionalization model of divergence, the biological functions of the ancestor could become partitioned between the two paralogs. Alternatively, in the presence of positive selection pressure one copy maintains the ancestral expression pattern, while the other one acquires a new expression pattern, and perhaps a new function. Possibly, the chance to evolve paralogs with new functions is even higher when multiple rounds of duplication of the ancestral gene succeed over evolutionary time. Accordingly, regulatory divergence in the form of expression pattern changes appears very frequently when comparing genes derived following a duplication event, both within and between metazoan genomes [51–58]. For instance, members of multigene families expanded rapidly as tandem duplicates displaying a multitude of highly diverse tissue-specific expression during embryogenesis of drosophilids [58–60]. In striking parallel to these observations, the *hbox12/pmar1/micro1* family of *P*. *lividus* combines members showing assorted spatiotemporal expression profiles, encompassing broad embryonic territories or only micromeres. The latter case pertains to *hbox12-09* and *-28*, whose transcription depends upon β-catenin signalling, similar to *pmar1*/*micro1* in other sea urchin species. Nevertheless, the mentioned genes cover incongruent genomic locations among sea urchin species, and there is total lack of conservation in their *cis*-regulatory elements, excluding a potential TCF/LEF-binding motif. From these findings, at least two interdependent conclusions can be inferred. First, the expression patterns among different sea urchin species is probably due, at least in part, to convergent evolution. Second, distinct selection pressures may have acted independently on each species to establish the current *hbox12/pmar1/micro1* gene copies. An explanation for the fact that these genes have different chromosomal positions among sea urchin species is that they underwent genomic rearrangement or, alternatively, they may have arisen by independent duplications. Whatever process changed the chromosomal disposition of these genes, it must have taken place rather rapidly on an evolutionary scale, since *L*. *variegatus*, *S*. *purpuratus*, and *P*. *lividus* diverged from a common ancestor roughly 30–50 million years ago, while the last two species diverged less than 20 million years ago [61–63].

In the case of *P*. *lividus*, it seems reasonable to suggest that the evolution process could also have involved transposition of single gene units to new physical locations in the genome, where subsequent waves of duplication would have led to expansion of the *hbox12/pmar1/micro1* family. A similar pattern of duplication and transposition events has been postulated to explain the evolution of various multigene families, such as those of the histone, TTY2, and vertebrate immune system genes [64–66]. Moreover, the occurrence of transposition and inversion episodes is supported by the different gene orientations in the DNA strands, and by the presence of several transposon relic sequences in the genomic regions neighbouring the *hbox12/pmar1/micro1* genes (not shown).

It is difficult to judge at this stage how many *hbox12/pmar1/micro1* family members represent functional genes. Indeed, although the presence of transcripts has been demonstrated in the developing embryo by qRT-PCR (Fig 3), we cannot rule out the possibility that some of the copies are pseudogenes. The transition between functional gene and pseudogene is usually gradual, and at the initial stages, the gene may continue to be transcribed. We noted that the predicted open reading frames of the four genes broadly transcribed in time and embryo territories are disrupted by premature translation termination codons (not shown). This observation may simply reflect artefactual mutations introduced during the sequencing procedure. Alternatively, it could be interpreted as decay of the functional sequence, and perhaps it supports the idea that these copies are indeed pseudogenes. Intriguingly, the lack of a regular ORF may also imply that these members of the *hbox12/pmar1/micro1* gene family function at the RNA level rather than encoding a protein product. In this regard, current literature of biosciences is dominated by examples of transcriptional units that, although lacking protein-coding capacity, are able to produce long functional RNAs [67].

### Copy number variation of the hbox12/pmar1/micro1 family

We show that *hbox12/pmar1/micro1* exists in the *P*. *lividus* genome as an extensive gene family exhibiting multiallelic copy number variation (CNV). CNV has been commonly associated with animal genomic portions containing gene families evolved by segmental duplication, which specifically refers to duplication of DNA fragments of at least 1 Kb [68,69]. The best characterized examples pertain to mammalian genes, such as human genes for olfactory receptors [70], the major histocompatibility complex class III and β-defensin antimicrobial gene clusters [71,72], genes at the amylase locus [73], and the paired-like homebox *RHOXF2* genes [74]. The *hbox12/pmar1/micro1* example adds to this list, representing the first report of CNV of a regulatory gene family of an invertebrate ambulacrarian organism.

Evidence in plants and case reports involving human patients have suggested that increasing the copy number of regulatory genes could modify phenotypes affecting, either positively or negatively, the expression level of target genes [75–78]. Conversely, a recent study highlighted substantial changes in the mRNA abundance of *pmar1* among embryo populations derived from mating of six male and female *S*. *purpuratus* individuals in every combination, but these incongruences had no detectable impact on the expression of known downstream genes involved in the specification of the micromere lineage [79]. The potential relationship between CNV of the *hbox12/pmar1/micro1* family and phenotypic variation deserves to be evaluated in similar large-scale studies in *P*. *lividus*, first requiring the identification of the direct target genes controlled by the various member of the family.

Another important consequence of CNV is that it could provide a mechanism for acquiring novel functions, and therefore phenotypes, through the occurrence of subsequent mutations of the different copies. For example, evolution of polychromatic color vision in primates resulted from sequence divergence within the *opsin* multicopy gene family [80,81], and structural variation of this visual pigment gene cluster affects red-green color discrimination capacity [82,83]. Similarly, it could be hypothesized that CNV at the *hbox12/pmar1/micro1* family may have provided a genetic substrate for the functional diversification of the *hbox12-a-like* and *pmar1/micro1-like* genes (see below).

### The hbox12-a-like and pmar1/micro1-like subfamilies

Our findings clearly indicate that in *P*. *lividus* the expression of the genes belonging to the *hbox12-a-like* and *pmar1/micro1-like* subfamilies is respectively restricted to animal blastomeres and micromeres of the early embryo. Equivalent, albeit indirect, findings were reported from studies in other sea urchin species. For example, *L*. *variegatus* micromere-less embryos assessed by qPCR were found to express *pmar1* at a reduced, although comparable, level with respect to intact embryos, indirectly revealing the expression of one or more members of the *hbox12-a-like* subfamily [84]. Indeed, given the remarkable conservation in the coding sequence within the *hbox12/pmar1/micro1* family, it is reasonable to presume that the oligonucleotide primers used in this study did not meet the required specificity to distinguish transcripts from the two subfamilies.

Another pertinent example comes from studies in *H*. *pulcherrimus* embryos, whereas a combination of whole mount in situ hybridization (WMISH), Northern blot hybridization using RNA extracted from blastomeres fractionated from 16-/32-cell stage embryos, and RT-PCR assays revealed two distinct and reproducible spatial expression domains of *micro1* expression, involving both the micromere and part of the mesomere lineages of 16-/32-cell stage embryos [22]. Similarly, and in accordance with unpublished evidence obtained during the optimization of our WMISH protocol, WMISH in *P*. *lividus* embryos at the early blastula stage clearly highlighted the apparent accumulation of *hbox12/pmar1/micro1* transcripts in broad spatial embryonic sectors, likely derived from both micromeres and animal blastomeres of earlier developmental stages [40]. In light of the findings described in the current study, we assume that cross-hybridization of the probe with transcripts from both the *hbox12-a-like* and *pmar1/micro1-like* subfamilies occurred during WMISH experiments in both the sea urchin species mentioned.

Systematic identification, as well as expression and *cis*-regulatory analysis of all the *hbox12/pmar1/micro1* genes from various sea urchin species will definitively clarify whether their transcripts are ubiquitous, circumscribed to certain embryonic sectors, or expressed at specific developmental stages.

In spite of the heterogeneity of their spatial expression profiles, the known *hbox12/pmar1/micro1* genes exhibit overt similarity in the coding sequences across distinct sea urchin species. This is not surprising, because the majority of duplicated genes with conserved coding sequences tend to rapidly acquire divergent expression pattern in organisms as diverse as yeast, humans, and plants [85–87]. A change in the spatial expression of duplicated genes coding for transcription factors could result in functional outcomes because most, if not all, transcription regulators do not work alone, but engage in complex interactions with other partners that may be differentially distributed among distinct cell types. Consistently with this, and assuming the obvious difference in the nuclear environment of animal blastomeres and micromeres of the early sea urchin embryo, it is possible that the different spatial transcription profiles of the genes belonging to the *hbox12-a-like* and *pmar1/micro1* subfamilies could account for divergent functions. In particular, the *hbox12-a-like* genes would be conjointly involved in the dorsal/ventral gene regulatory network, while the *pmar1/micro1-like* genes would be required to program the specification of the micromere lineage.

The reported difference in the transcriptional level of the two subfamilies could provide an additional layer of functional diversification, as change in the absolute amount of the corresponding transcription factors may affect selection of subset of target sequences with differential binding affinity. In this regard, it was clear to us from many foregoing experiments that the severity of the morphological effects inflicted by overexpression of *hbox12-a* correlated with the proportional increase of the injected *hbox12-a* mRNA. In particular, when ectopically expressed at sub-physiological dosages, *hbox12-a* partially affects *nodal* expression and exerts a reproducible phenotype in a fraction (up to 50%) of the injected embryos [14]. By contrast, when higher amounts of *hbox12-a* mRNA are injected, nearly all embryos (n>1000) observed from the mesenchyme blastula stage onwards undergo massive epithelial-mesenchymal transition (not shown). It could be argued that, over a threshold of concentration, the ectopic expression of *hbox12-a* might have unspecifically phenocopied the developmental aberrations described in *S*. *purpuratus* and *H*. *pulcherrimus* embryos misexpressing *pmar1* and *micro1*, respectively [21,23].

Significantly, a comparable effect was also observed in *P*. *lividus* embryos following injection of a synthetic mRNA corresponding to a not well-defined member of the *hbox12/pmar1/micro1* family, at doses above or equal to 5 μg/ml [40]. By contrast, the same transcript did not produce detectable effects on dorsal-ventral polarization when injected in the developing zygote at concentration below 5 μg/ml [40]. Given the partial penetrance of the phenotype inflicted by ubiquitous *hbox12-a* overexpression [14], in the absence of statistics and analysis of *nodal* and/or *nodal*-dependent gene transcription at early stages, these results could likely reflect a limited effect on dorsal-ventral polarization, followed by recovery during embryogenesis.

In conclusion, our study provides the first comprehensive analysis of the *hbox12/pmar1/micro1* multigene family. Members of this family appear to have evolved separately in different indirectly developing sea urchin species, where they fulfil distinct functions. Future work in this direction will benefit from investigating this gene family in further echinoid species.

## Supporting information

S1 Table List of gene-specific oligonucleotides used in the quantitative PCR and RT-PCR. (DOC)

S1 Fig Multiple ClustalW alignment of the homeodomain sequences deduced for the *hbox12/pmar1/micro1* genes of *P*. *lividus*. Identical residues in all of the aminoacid sequences are marked by asterisks. Dashes represent the gaps inserted for maximal alignment, while stretches of dashes located either at the COOH- or NH2-terminal end of hbox12-19, -21, and -27 indicate lack of protein sequence.
(TIF)

S2 Fig Pairwise comparisons of the amino acid sequences deduced from the indicated *hbox12/pmar1/micro1* family members. The names of the sequences identified in this study are indicated in black, while those of the proteins previously described [40] are shown in blue. For each alignment, identical residues are indicated by asterisks, while differences are highlighted in red. The sequences of Hbox12-5 and -27 are incomplete at the COOH terminus.
(TIF)

S3 Fig Temporal expression of *P*. *lividus hbox12/pmar1/micro1* genes investigated by qPCR surveys. The expression profile of every gene is displayed individually, together with standard errors of the mean between replicates for each developmental stage assayed. The developmental stages are as follows: Egg, unfertilized egg; 16-cell, fourth cleavage embryo; VEB, very early blastula; MB, mesenchyme blastula; LG, late gastrula.
(TIF)

S4 Fig Comparison of *hbox12/pmar1/micro1* loci across sea urchin species. The mVISTA software package was used to determine evolutionary conserved regions among the two sea urchin species indicated, using *pmar1b* from *S*. *purpuratus* as the reference sequence. Each graph show a pairwise alignment with the extent of sequence identity plotted on the Y-axis against the indicated sequence. The grey arrow below each graph shows the extent of sequence used, while filled portions indicate conservation (>70% over 100 bp) of either exons (labeled in blue) or noncoding sequences (pink). Note that significant sequence similarity is found exclusively in the protein coding regions across the two species.
(TIF)

S5 Fig Diagrammatic representation of the *hbox12/pmar1/micro1* genes from *P*. *lividus* (*hbox12-09* and -*28*) and *S*. *purpuratus* (*pmar1a-d*), indicating the relative location of the predicted TCF/LEF consensus binding sites. The motifs mapping on the sense and antisense DNA strand are represented respectively above and below the diagram. Pink shading indicates conservation in the relative position of a TCF/LEF motif in 5 out of 6 genes.
(TIF)

## References

[1] Ettensohn CA. Encoding anatomy: developmental gene regulatory networks and morphogenesis. Genesis 2013; 51:383–409. 10.1002/dvg.22380 23436627

[2] Martik ML, Lyons DC, McClay DR. Developmental gene regulatory networks in sea urchins and what we can learn from them. F1000Res 2016; 5. 10.12688/f1000research.7381.1 PMC4765714 26962438

[3] Parfitt DE, Shen MM. From blastocyst to gastrula: gene regulatory networks of embryonic stem cells and early mouse embryogenesis. Philos Trans R Soc Lond B Biol Sci 2014; 369. 10.1098/rstb.2013.0542 PMC4216465 25349451

[4] Satou Y, Imai KS. Gene regulatory systems that control gene expression in the Ciona embryo. Proc Jpn Acad Ser B Phys Biol Sci 2015; 91:33–51. 10.2183/pjab.91.33 25748582 PMC4406867

[5] Hinman VF, Nguyen AT, Cameron RA, Davidson EH. Developmental gene regulatory network architecture across 500 million years of echinoderm evolution. Proc Natl Acad Sci USA 2003; 100:13356–61. 10.1073/pnas.2235868100 14595011 PMC263818

[6] McCauley BS, Weideman EP, Hinman VF. A conserved gene regulatory network subcircuit drives different developmental fates in the vegetal pole of highly divergent echinoderm embryos. Dev Biol 2012; 340:200–8. 10.1016/j.ydbio.2009.11.020 19941847

[7] Peter IS, Davidson EH. Evolution of gene regulatory networks controlling body plan development. Cell 2011; 144:970–85. 10.1016/j.cell.2011.02.017 21414487 PMC3076009

[8] Prud’homme B, Gompel N, Rokas A, Kassner VA, Williams TM, Yeh SD, et al Repeated morphological evolution through cis-regulatory changes in a pleiotropic gene. Nature 2006; 440:1050–3. 10.1038/nature04597 16625197

[9] Royo JL, Maeso I, Irimia M, Gao F, Peter IS, Lopes CS, et al Transphyletic conservation of developmental regulatory state in animal evolution. Proc Natl Acad Sci USA 2011; 108:14186–91. 10.1073/pnas.1109037108 21844364 PMC3161536

[10] Zhang JZ. Evolution by gene duplication: an update. Trends Ecol Evol 2003; 18:292–8.

[11] Lynch M. The origins of genome architecture Sunderland: Sinauer Associates; 2007.

[12] Di Bernardo M, Russo R, Oliveri P, Melfi R, and Spinelli G. Homeobox-containing gene transiently expressed in a spatially restricted pattern in the early sea urchin embryo. Proc Natl Acad Sci USA 1995; 92:8180–4. 7667265 10.1073/pnas.92.18.8180 PMC41120

[13] Cavalieri V, Di Bernardo M, Anello L, and Spinelli G. cis-Regulatory sequences driving the expression of the Hbox12 homeobox-containing gene in the presumptive aboral ectoderm territory of the Paracentrotus lividus sea urchin embryo. Dev Biol 2008; 321:455–69. 10.1016/j.ydbio.2008.06.006 18585371

[14] Cavalieri V, Spinelli G. Early asymmetric cues triggering the dorsal/ventral gene regulatory network of the sea urchin embryo. Elife 2014; 3:e04664 10.7554/eLife.04664 25457050 PMC4273433

[15] Cavalieri V, Spinelli G. Ectopic hbox12 Expression Evoked by Histone Deacetylase Inhibition Disrupts Axial Specification of the Sea Urchin Embryo. PLoS One 2015; 10:e0143860 10.1371/journal.pone.0143860 26618749 PMC4664418

[16] Cavalieri V, Spinelli G. Symmetry Breaking and Establishment of Dorsal/Ventral Polarity in the Early Sea Urchin Embryo. Symmetry 2015; 7(4): 1721–33.

[17] Duboc V, Röttinger E, Besnardeau L, and Lepage T. Nodal and BMP2/4 signaling organizes the oral-aboral axis of the sea urchin embryo. Dev Cell 2004; 6:397–410. 15030762 10.1016/s1534-5807(04)00056-5

[18] Duboc V, Lapraz F, Saudemont A, Bessodes N, Mekpoh F, Haillot E, et al Nodal and BMP2/4 pattern the mesoderm and endoderm during development of the sea urchin embryo. Development 2010; 137:223–35. 10.1242/dev.042531 20040489

[19] Flowers VL, Courteau GR, Poustka AJ, Weng W, Venuti JM. Nodal/activin signaling establishes oral-aboral polarity in the early sea urchin embryo. Dev Dyn 2004; 231:727–40. 10.1002/dvdy.20194 15517584

[20] Materna SC, Ransick A, Li E, Davidson EH. Diversification of oral and aboral mesodermal regulatory states in pregastrular sea urchin embryos. Dev Biol 2013; 375:92–104. 10.1016/j.ydbio.2012.11.033 23261933 PMC3570723

[21] Oliveri P, Carrick DM, Davidson EH. A regulatory gene network that directs micromere specification in the sea urchin embryo. Dev Biol 2002; 246:209–28. 10.1006/dbio.2002.0627 12027443

[22] Kitamura K, Nishimura Y, Kubotera N, Higuchi Y, Yamaguchi M. Transient activation of the micro1 homeobox gene family in the sea urchin (Hemicentrotus pulcherrimus) micromere. Dev Genes Evol 2002; 212:1–10. 10.1007/s00427-001-0202-3 11875651

[23] Nishimura Y, Sato T, Morita Y, Yamazaki A, Akasaka K, Yamaguchi M. Structure, regulation, and function of micro1 in the sea urchin Hemicentrotus pulcherrimus. Dev Genes Evol 2004; 214:525–36. 10.1007/s00427-004-0442-0 15480758

[24] Wu SY, McClay DR. The Snail repressor is required for PMC ingression in the sea urchin embryo. Development 2007; 134:1061–70. 10.1242/dev.02805 17287249 PMC3045531

[25] Ettensohn CA, Kitazawa C, Cheers MS, Leonard JD, Sharma T. Gene regulatory networks and developmental plasticity in the early sea urchin embryo: alternative deployment of the skeletogenic gene regulatory network. Development 2007; 134:3077–87. 10.1242/dev.009092 17670786

[26] Oliveri P, Davidson EH, McClay DR. Activation of pmar1 controls specification of micromeres in the sea urchin embryo. Dev Biol 2003; 258:32–43. 12781680 10.1016/s0012-1606(03)00108-8

[27] Yamazaki A, Kawabata R, Shiomi K, Amemiya S, Sawaguchi M, Mitsunaga-Nakatsubo K, et al The micro1 gene is necessary and sufficient for micromere differentiation and mid/hindgut-inducing activity in the sea urchin embryo. Dev Genes Evol 2005; 215:450–9. 10.1007/s00427-005-0006-y 16078091

[28] Revilla-i-Domingo R, Oliveri P, Davidson EH. A missing link in the sea urchin embryo gene regulatory network: hesC and the double-negative specification of micromeres. Proc Natl Acad Sci USA 2007; 104:12383–8. 10.1073/pnas.0705324104 17636127 PMC1941478

[29] Di Bernardo M, Castagnetti S, Bellomonte D, Oliveri P, Melfi R, Palla F, et al Spatially restricted expression of PlOtp, a Paracentrotus lividus orthopedia-related homeobox gene, is correlated with oral ectodermal patterning and skeletal morphogenesis in late-cleavage sea urchin embryos. Development 1999; 126:2171–9. 10207142 10.1242/dev.126.10.2171

[30] Cavalieri V, Melfi R, and Spinelli G. The Compass-like locus, exclusive to the ambulacrarians, encodes a chromatin insulator binding protein in the sea urchin embryo. PLoS Genet 2013; 9:e1003847 10.1371/journal.pgen.1003847 24086165 PMC3784565

[31] Cavalieri V, Melfi R, and Spinelli G. Promoter activity of the sea urchin (Paracentrotus lividus) nucleosomal H3 and H2A and linker H1 α-histone genes is modulated by enhancer and chromatin insulator. Nucleic Acids Res 2009; 37:7407–15. 10.1093/nar/gkp859 19843609 PMC2794192

[32] Baiamonte E, Spinelli G, Maggio A, Acuto S, Cavalieri V. The Sea Urchin sns5 Chromatin Insulator Shapes the Chromatin Architecture of a Lentivirus Vector Integrated in the Mammalian Genome. Nucleic Acid Ther 2016; 26:318–26. 10.1089/nat.2016.0614 27248156

[33] Wang DGW, Britten RJ, Davidson EH. Maternal and embryonic provenance of a sea urchin embryo transcription factor, SpZ12-1. Mol Marine Biol Biotechnol 1995; 4:148–53. 7773332

[34] Materna SC, Nam J, Davidson EH. High accuracy, high-resolution prevalence measurement for the majority of locally expressed regulatory genes in early sea urchin development. Gene Expr Patterns 2010; 10:177–84. 10.1016/j.gep.2010.04.002 20398801 PMC2902461

[35] Cavalieri V, Guarcello R, and Spinelli G. Specific expression of a TRIM-containing factor in ectoderm cells affects the skeletal morphogenetic program of the sea urchin embryo, Development 2011; 138:4279–90. 10.1242/dev.066480 21896632

[36] Cavalieri V, Di Bernardo M, Spinelli G. Regulatory sequences driving expression of the sea urchin Otp homeobox gene in oral ectoderm cells. Gene Expr Patterns 2007; 7:124–30. 10.1016/j.modgep.2006.06.001 16843737

[37] Cavalieri V, Di Bernardo M, and Spinelli G. Functional studies of regulatory genes in the sea urchin embryo. Methods Mol Biol 2009; 518:175–88. 10.1007/978-1-59745-202-1_13 19085138

[38] Logan CY, Miller JR, Ferkowicz MJ, McClay DR. Nuclear beta-catenin is required to specify vegetal cell fates in the sea urchin embryo. Development 1999; 126:345–57. 9847248 10.1242/dev.126.2.345

[39] Röttinger E, Besnardeau L, Lepage T. A Raf/MEK/ERK signaling pathway is required for development of the sea urchin embryo micromere lineage through phosphorylation of the transcription factor Ets. Development 2004; 131:1075–87. 10.1242/dev.01000 14973284

[40] Haillot E, Molina MD, Lapraz F, Lepage T. The Maternal Maverick/GDF15-like TGF-β Ligand Panda Directs Dorsal-Ventral Axis Formation by Restricting Nodal Expression in the Sea Urchin Embryo. PLoS Biol 2015; 13(9):e1002247 10.1371/journal.pbio.1002247 26352141 PMC4564238

[41] Ettensohn CA, Illies MR, Oliveri P, De Jong DL. Alx1, a member of the Cart1/Alx3/Alx4 subfamily of Paired-class homeodomain proteins, is an essential component of the gene network controlling skeletogenic fate specification in the sea urchin embryo. Development 2003; 130:2917–28. 12756175 10.1242/dev.00511

[42] Dereeper A, Guignon V, Blanc G, Audic S, Buffet S, Chevenet F, et al Phylogeny.fr: robust phylogenetic analysis for the non-specialist. Nucleic Acids Res 2008; 36:W465–9. 10.1093/nar/gkn180 18424797 PMC2447785

[43] Wikramanayake AH, Peterson R, Chen J, Huang L, Bince JM, McClay DR, et al Nuclear beta-catenin-dependent Wnt8 signaling in vegetal cells of the early sea urchin embryo regulates gastrulation and differentiation of endoderm and mesodermal cell lineages. Genesis 2004; 39(3):194–205. 10.1002/gene.20045 15282746

[44] Fagotto F, Funayama N, Gluck U, Gumbiner BM. Binding to cadherins antagonizes the signaling activity of beta-catenin during axis formation in Xenopus. J Cell Biol 1996; 132:1105–14. 8601588 10.1083/jcb.132.6.1105 PMC2120760

[45] Sanson B, White P, Vincent JP. Uncoupling cadherin-based adhesion from wingless signalling in Drosophila. Nature 1996; 383:627–30. 10.1038/383627a0 8857539

[46] Brudno M, Do CB, Cooper GM, Kim MF, Davydov E, NISC Comparative Sequencing Program, et al LAGAN and Multi-LAGAN: efficient tools for large-scale multiple alignment of genomic DNA. Genome Res 2003; 13:721–31. 10.1101/gr.926603 12654723 PMC430158

[47] Mayor C, Brudno M, Schwartz JR, Poliakov A, Rubin EM, Frazer KA, et al VISTA: visualizing global DNA sequence alignments of arbitrary length. Bioinformatics 2000; 16:1046–7. 11159318 10.1093/bioinformatics/16.11.1046

[48] Hoekstra HE, Coyne JA. The locus of evolution: evo devo and the genetics of adaptation. Evolution 2007; 61:995–1016. 10.1111/j.1558-5646.2007.00105.x 17492956

[49] Teichmann SA, Babu MM. Gene regulatory network growth by duplication. Nat Genet 2004; 36:492–6. 10.1038/ng1340 15107850

[50] Lynch M, Conery JS. The evolutionary fate and consequences of duplicate genes. Science 2000; 290:1151–5. 11073452 10.1126/science.290.5494.1151

[51] Assis R, Bachtrog D. Neofunctionalization of young duplicate genes in Drosophila. Proc Natl Acad Sci USA 2013; 110:17409–14. 10.1073/pnas.1313759110 24101476 PMC3808614

[52] Castillo-Davis CI, Hartl DL, Achaz G. cis-Regulatory and protein evolution in orthologous and duplicate genes. Genome Res 2004; 14:1530–6. 10.1101/gr.2662504 15256508 PMC509261

[53] Force A, Lynch M, Pickett FB, Amores A, Yan YL, Postlethwait J. Preservation of duplicate genes by complementary, degenerative mutations. Genetics 1999; 151:1531–45. 10101175 10.1093/genetics/151.4.1531 PMC1460548

[54] Ohno S. Evolution by gene duplication Berlin: Springer-Verlag; 1970.

[55] Gu Z, Rifkin SA, White KP, Li WH. Duplicate genes increase gene expression diversity within and between species. Nat Genet 2004; 36:577–9. 10.1038/ng1355 15122255

[56] Ha M, Kim ED, Chen ZJ. Duplicate genes increase expression diversity in closely related species and allopolyploids. Proc Natl Acad Sci USA 2009; 106:2295–300. 10.1073/pnas.0807350106 19168631 PMC2650150

[57] Katju V. To the beat of a different drum: determinants implicated in the asymmetric sequence divergence of Caenorhabditis elegans paralogs. BMC Evol Biol 2013; 13:73 10.1186/1471-2148-13-73 23530733 PMC3637608

[58] Tanaka K, Diekmann Y, Hazbun A, Hijazi A, Vreede B, Roch F, et al Multispecies Analysis of Expression Pattern Diversification in the Recently Expanded Insect Ly6 Gene Family. Mol Biol Evol 2015; 32:1730–47. 10.1093/molbev/msv052 25743545 PMC4476152

[59] Fradkin LG, Kamphorst JT, DiAntonio A, Goodman CS, Noordermeer JN. Genome-wide analysis of the Drosophila tetraspanins reveals a subset with similar function in the formation of the embryonic synapse. Proc Natl Acad Sci USA 2002; 99:13663–8. 10.1073/pnas.212511099 12370414 PMC129735

[60] Patel MV, Hallal DA, Jones JW, Bronner DN, Zein R, Caravas J, et al Dramatic expansion and developmental expression diversification of the methuselah gene family during recent Drosophila evolution. J Exp Zool Part B Mol Dev Evol 2012; 318: 368–87. 10.1002/jez.b.22453 22711569

[61] Cameron RA, Samanta M, Yuan A, He D, Davidson E. SpBase: the sea urchin genome database and web site. Nucleic Acids Res 2009; 37:D75075–4. 10.1093/nar/gkn887 PMC2686435 19010966

[62] Littlewood DT., Smith AB. A combined morphological and molecular phylogeny for sea urchins (Echinoidea: Echinodermata). Philos Trans R Soc Lond B Biol Sci 1995, 347:213–34. 10.1098/rstb.1995.0023 7746863

[63] Smith AB, Pisani D, Mackenzie-Dodds JA, Stockley B, Webster BL, Littlewood DT. Testing the molecular clock: molecular and paleontological estimates of divergence times in the Echinoidea (Echinodermata). Mol Biol Evol 2006; 23:1832–51. 10.1093/molbev/msl039 16777927

[64] Eirín-López JM, González-Tizón AM, Martínez A, Méndez J. Birth-and-death evolution with strong purifying selection in the histone H1 multigene family and the origin of orphon H1 genes. Mol Biol Evol 2004; 21:1992–2003. 10.1093/molbev/msh213 15254261

[65] Makrinou E, Fox M, Lovett M, Haworth K, Cameron JM, Taylor K, et al TTY2: a multicopy Y-linked gene family. Genome Res 2001; 11:935–45. 10.1101/gr.175901 11381023 PMC311066

[66] Sitnikova T, Nei M. Evolution of immunoglobulin kappa chain variable region genes in vertebrates. Mol Biol Evol 1998; 15:50–60. 9491604 10.1093/oxfordjournals.molbev.a025846

[67] Quinn JJ, Chang HY. Unique features of long non-coding RNA biogenesis and function. Nat Rev Genet 2016; 17:47–62. 10.1038/nrg.2015.10 26666209

[68] Feuk L, Carson AR, and Scherer SW. Structural variation in the human genome. Nat Rev Genet 2006; 7:85–97. 10.1038/nrg1767 16418744

[69] Freeman JL, Perry GH, Feuk L, Redon R, McCarroll SA, Altshuler DM, et al Copy number variation: new insights in genome diversity. Genome Res 2006; 16:949–61. 10.1101/gr.3677206 16809666

[70] Trask BJ, Friedman C, Martin-Gallardo A, Rowen L, Akinbami C, Blankenship J, et al Members of the olfactory receptor gene family are contained in large blocks of DNA duplicated polymorphically near the ends of human chromosomes. Hum Mol Genet 1998; 7:13–26. 9384599 10.1093/hmg/7.1.13

[71] Ghanem N, Uring-Lambert B, Abbai M, Hauptmann G, Lefranc MP, and Lefranc G. Polymorphism of MHC class III genes: Definition of restriction fragment linkage groups and evidence for frequent deletions and duplications. Hum Genet 1988; 79:209–18. 2900212 10.1007/BF00366239

[72] Hollox EJ, Armour JAL, and Barber JCK. Extensive normal copy number variation of a β-Defensin antimicrobial-gene cluster. Am J Hum Genet 2003; 73:591–600. 10.1086/378157 12916016 PMC1180683

[73] Groot PC, Mager WH, and Frants RF. Interpretation of polymorphic DNA patterns in the human α-amylase multigene family. Genomics 1991; 10:779–85. 1679752 10.1016/0888-7543(91)90463-o

[74] Niu AL, Wang YQ, Zhang H, Liao CH, Wang JK, Zhang R, et al Rapid evolution and copy number variation of primate RHOXF2, an X-linked homeobox gene involved in male reproduction and possibly brain function. BMC Evol Biol 2011; 11:298 10.1186/1471-2148-11-298 21988730 PMC3214919

[75] Cook DE, Lee TG, Guo X, Melito S, Wang K, Bayless AM, et al Copy number variation of multiple genes at Rhg1 mediates nematode resistance in soybean. Science 2012; 338:1206–9. 10.1126/science.1228746 23065905

[76] Somerville MJ, Mervis CB, Young EJ, Seo EJ, del Campo M, Bamforth S, et al Severe Expressive-Language Delay Related to Duplication of the Williams–Beuren Locus. N Engl J Med 2005; 353:1694–701. 10.1056/NEJMoa051962 16236740 PMC2893213

[77] Lee JA, Madrid RE, Sperle K, Ritterson CM, Hobson GM, Garbern J, et al Spastic paraplegia type 2 associated with axonal neuropathy and apparent PLP1 position effect. Ann Neurol 2006; 59:398–403. 10.1002/ana.20732 16374829

[78] Linzmeier RM, Ganz T. Human defensin gene copy number polymorphisms: comprehensive analysis of independent variation in alpha- and beta-defensin regions at 8p22-p23. Genomics 2005; 86(4):423–30. 10.1016/j.ygeno.2005.06.003 16039093

[79] Garfield DA, Runcie DE, Babbitt CC, Haygood R, Nielsen WJ, Wray GA. The impact of gene expression variation on the robustness and evolvability of a developmental gene regulatory network. PLoS Biol 2013; 11(10):e1001696 10.1371/journal.pbio.1001696 24204211 PMC3812118

[80] Nei M, Zhang J, Yokoyama S. Color vision of ancestral organisms of higher primates. Mol Biol Evol 1997; 14: 611–8. 9190062 10.1093/oxfordjournals.molbev.a025800

[81] Yokoyama S, Radlwimmer FB. The molecular genetics of red and green color vision in mammals. Genetics 1999; 153:919–32. 10511567 10.1093/genetics/153.2.919 PMC1460773

[82] Jagla WM, Jagle H, Hayashi T, Sharpe LT, Deeb SS. The molecular basis of dichromatic color vision in males with multiple red and green visual pigment genes. Hum Mol Genet 2002; 11:23–32. 11772996 10.1093/hmg/11.1.23

[83] Neitz J, Neitz M, Kainz PM. Visual pigment gene structure and the severity of color vision defects. Science 1996; 274:801–4. 8864125 10.1126/science.274.5288.801

[84] Cheng X, Lyons DC, Socolar JE, McClay DR. Delayed transition to new cell fates during cellular reprogramming. Dev Biol 2014; 391:147–57. 10.1016/j.ydbio.2014.04.015 24780626 PMC4064802

[85] Gu Z, Cavalcanti A, Chen FC, Bouman P, Li WH. Extent of gene duplication in the genomes of Drosophila, nematode, and yeast. Mol Biol Evol 2002; 19:256–62. 11861885 10.1093/oxfordjournals.molbev.a004079

[86] Makova KD, and Li WH. Divergence in the spatial pattern of gene expression between human duplicate genes. Genome Res 2003; 13:1638–45. 10.1101/gr.1133803 12840042 PMC403737

[87] Blanc G, and Wolfe KH. Functional Divergence of Duplicated Genes Formed by Polyploidy during Arabidopsis Evolution. Plant Cell 2004; 16:1679–91. 10.1105/tpc.021410 15208398 PMC514153

