# Supplementary material for: Diversification of spatiotemporal expression and copy number variation of the echinoid hbox12/pmar1/micro1 multigene family
Source: PLoS One. 2017 Mar 28;12(3):e0174404. doi: 10.1371/journal.pone.0174404 (PMC5370098; doi:10.1371/journal.pone.0174404)
Supplement: S1 Table — (DOC) [file pone.0174404.s001.doc]

**S1 Table. List of gene-specific oligonucleotides used in the quantitative PCR and RT-PCR.**

| Target gene | Forward (F) and Reverse (R) sequences (5’ to 3’) | Length | Amplicon size (bp) | Figures in which oligonucleotide was used |
| --- | --- | --- | --- | --- |
| *hbox12-universal* | F: ACGTCTTCGTCGAGCATCTC  R: GCATGGTGCCTTTCGCTTACG | 20  21 | 124 | Fig. 2 |
| *otp* | F: CCTGTACTCATTCAACCAATC  R: GAATCAGATGTTAGGTGGAAAT | 21  22 | 181 | Fig. 2 |
| *cmpl* | F: CTGTATATTGATGGTTAGTCAA  R: ACCACAGTACATCTCACCGC | 22  20 | 163 | Fig. 2 |
| *hbox12-a* | F: TTGATGTCAGTAGACTTTCTCTCTCG  R: GTACGGATAATGACCCATCCAT | 26  22 | 135 | Figs 3, 4B, 7B, and S3 |
| *hbox12-29* | F: TTGATGTCAGTAGACTTTCTCTCTCG  R: GCTCTGCGATATACCTGGATGTA | 26  23 | 173 | Figs 3, 4B, 7B, and S3 |
| *hbox12-04** | F: TTGATGTCAGTAGACTTTCTCTCTCG  R: CAAAGTTCAAACAATGCCTAACG | 26  23 | 170 | Figs 3, 4B, and S3 |
| *hbox12-06* | F: AGACTTTCTCTCTCGTAGCAGCC  R: TCATATCACTCTGATAGGTCCATGAT | 23  26 | 228 | Figs 3, 4B, 7B, and S3 |
| *hbox12-09* | F: AGACTTTCTCTCTCGTAGCAGCC  R: CTGCGTTATACCTGGATGACTTG | 23  23 | 64 | Figs 3, 4B, 7B, and S3 |
| *hbox12-10* | F: AGACTTTCTCTCTCGTAGCAGCC  R: GAAGTTAACAAGTGAATCAAACAATGTC | 23  28 | 165 | Figs 3, 4B, 7B, and S3 |
| *hbox12-28* | F: AGACTTTCTCTCTCGTAGCAGCC  R: CGATTCCGACAAAACAGCATA | 23  21 | 196 | Figs 3, 4B, 7B, and S3 |
| *hbox12-12* | F: AGACTTTCTCTCTCGTAGCAGCC  R: AAATATCAACAGTGTCAATGAGTTCATC | 23  28 | 173 | Figs 3, 7B, and S3 |
| *hbox12-16* | F: TTGATGTCAGTAGACTTTCTCTCTCG  R: CGGTGTGTGCTCTCTTGGG | 26  19 | 113 | Figs 3, 4B, 7B, and S3 |
| *hbox12-17* | F: AGACTTTCTCTCTCGTAGCAGCC  R: AGTCAATGAGTTCACCAAACAATG | 23  23 | 222 | Figs 3, 4B, 7B, and S3 |
| *hbox12-19* | F: AGACTTTCTCTCTCGTAGCAGCC  R: GTATCAACAATGTCAATGAGTTCACC | 23  26 | 228 | Figs 3 and S3 |
| *hbox12-24* | F: TTGATGTCAGTAAACTTTCTCTCTCG  R: GACCCATCGGTGTGCGGT | 26  18 | 97 | Figs 3, 4B, 7B, and S3 |
| *alx* | F: GATTCATCAATCATCGCCGTGG  R: GGAAGGTTGGACTGGGGTGC | 22  20 | 138 | Figs 4B and 7B |
| *mbf-1*^§^ | F: ATGACACAGCCTGGAGCT  R: TACCAAGGAAGTGGGTGT | 18  18 | 102 | Figs 3, 4B, 7B, and S3 |
| *cyt-ox*^§^ | F: GTTGGGGTTAATCTAACATTCTTC  R: GAGGGTATAGGCATCTGGATAG | 24  22 | 93 | Figs 3, 4B, 7B, and S3 |
| *z12*^†^ | F: AGCGCCACACCAAAAGAAGTC  R: GGATGATAGACAGGGCTGTTTGGA | 21  24 | 93 | Fig. 3 |

^*^ The *hbox12-04* primer pair co-amplified segment of the *hbox12-04*, *-21* and *-25* genes.

^§^ The *H2A* histone modulator binding factor (*mbf-1*), *z12*, or a *cytochrome-oxidase* (*cyt-ox*) mRNA were used to normalize all data.

^†^ The *z12* mRNA was used to estimate the number of *hbox12* transcripts per embryo.
