# Supplementary figures and images for: Diversification of spatiotemporal expression and copy number variation of the echinoid hbox12/pmar1/micro1 multigene family
Source: PLoS One. 2017 Mar 28;12(3):e0174404. doi: 10.1371/journal.pone.0174404 (PMC5370098; doi:10.1371/journal.pone.0174404)

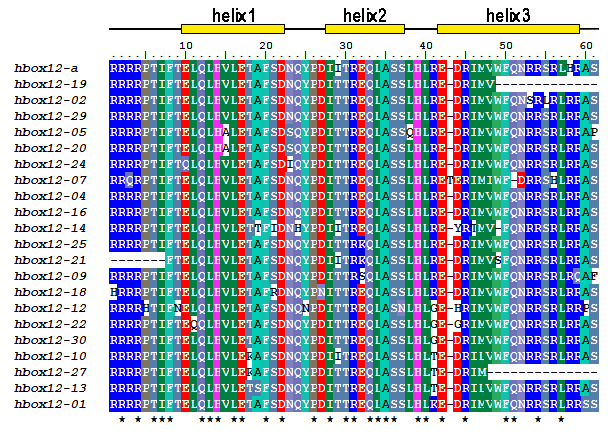

Supplement: S1 Fig — Identical residues in all of the aminoacid sequences are marked by asterisks. Dashes represent the gaps inserted for maximal alignment, while stretches of dashes located either at the COOH- or NH2-terminal end of hbox12-19, -21, and -27 indicate lack of protein sequence. (TIF) [file pone.0174404.s002.tif]

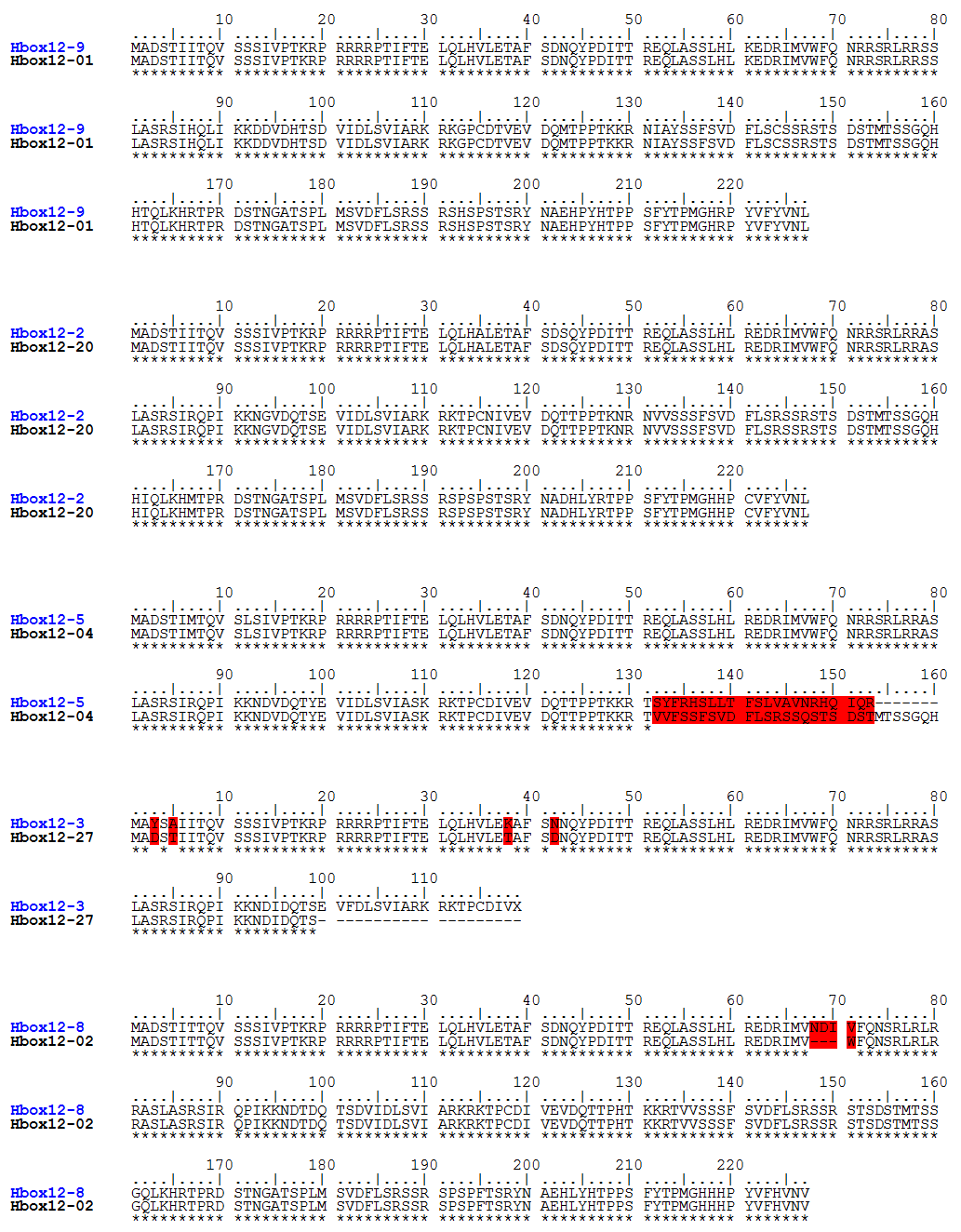

Supplement: S2 Fig — The names of the sequences identified in this study are indicated in black, while those of the proteins previously described [40] are shown in blue. For each alignment, identical residues are indicated by asterisks, while differences are highlighted in red. The sequences of Hbox12-5 and -27 are incomplete at the COOH terminus. (TIF) [file pone.0174404.s003.tif]

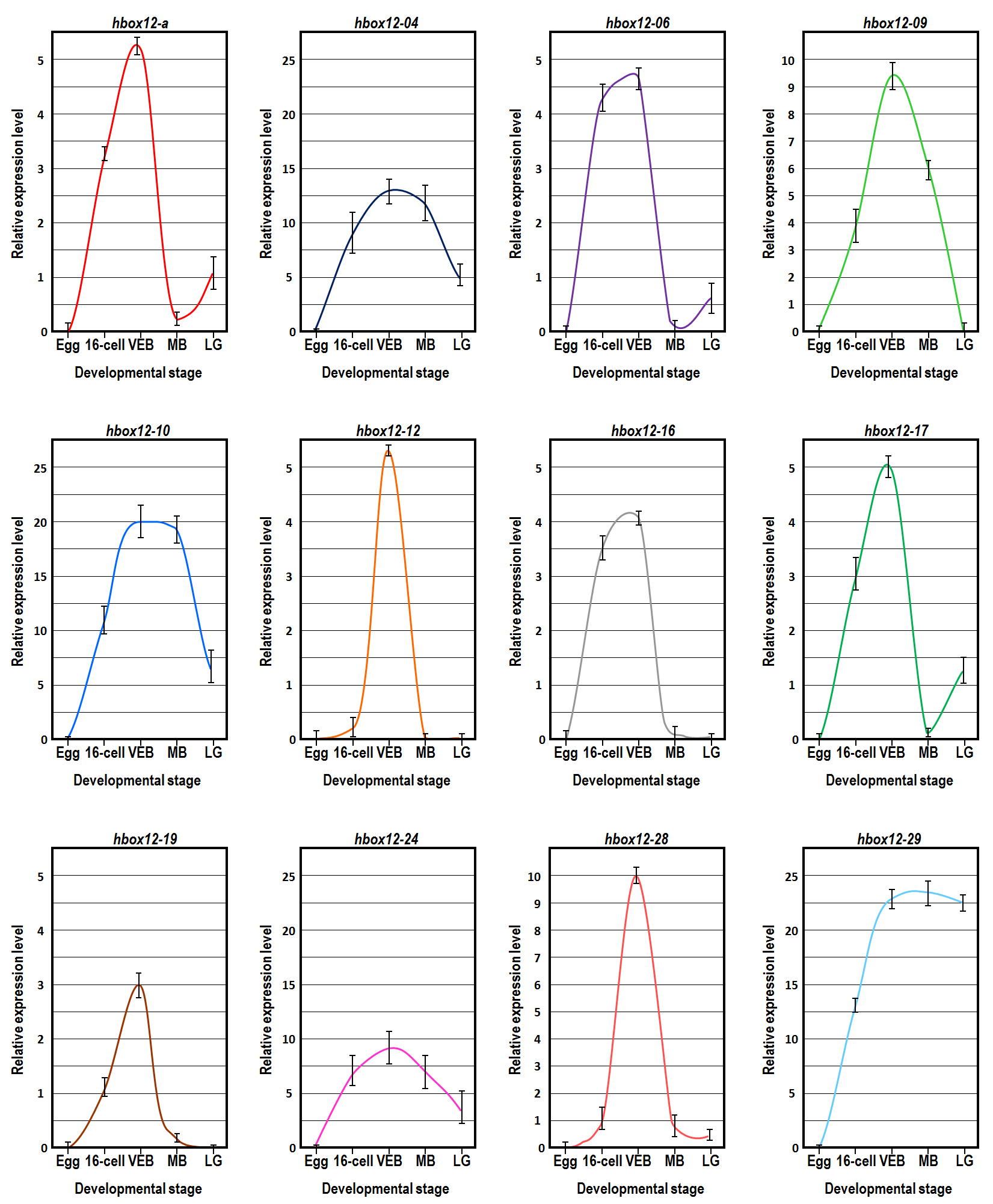

Supplement: S3 Fig — The expression profile of every gene is displayed individually, together with standard errors of the mean between replicates for each developmental stage assayed. The developmental stages are as follows: Egg, unfertilized egg; 16-cell, fourth cleavage embryo; VEB, very early blastula; MB, mesenchyme blastula; LG, late gastrula. (TIF) [file pone.0174404.s004.tif]

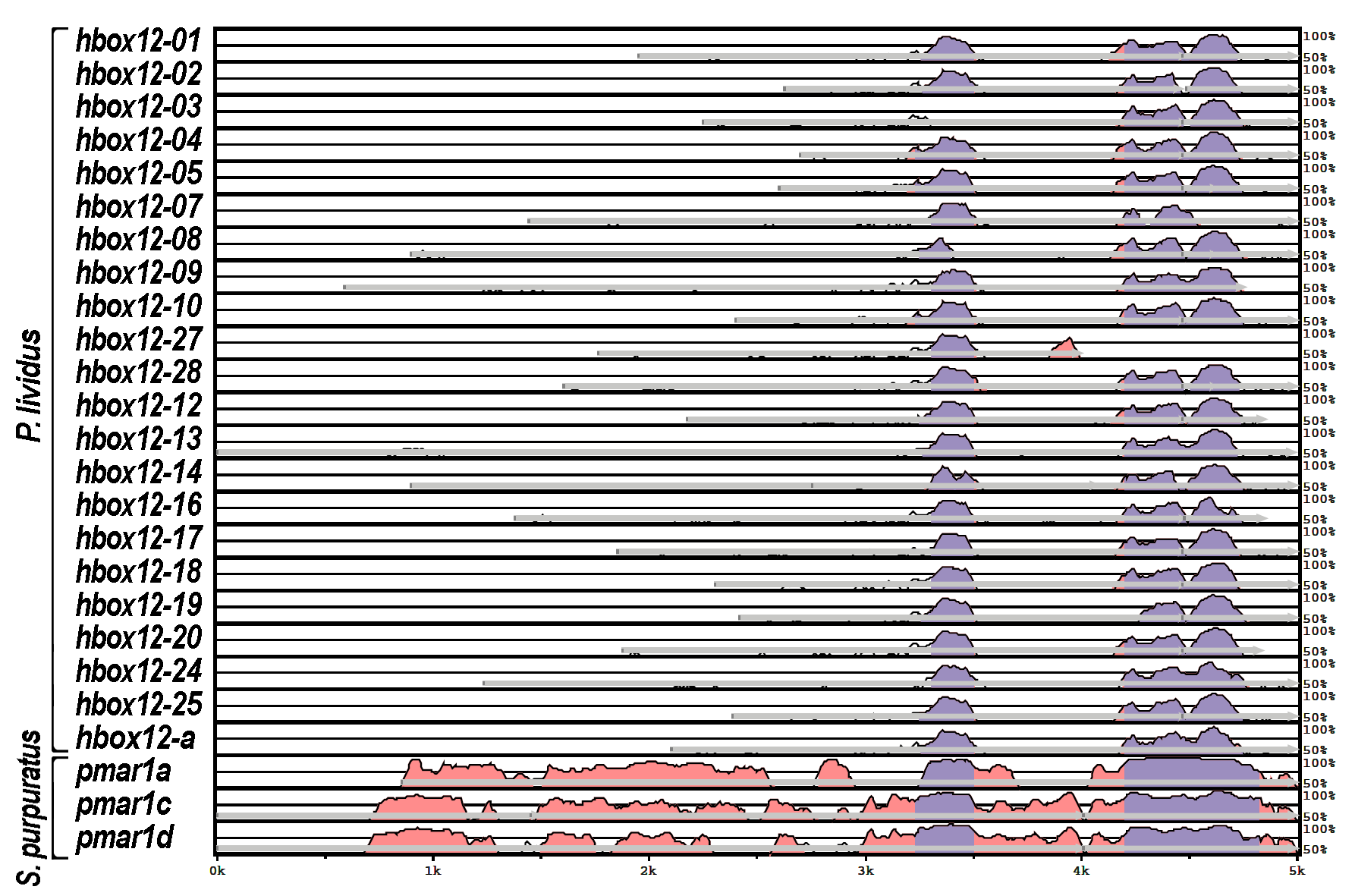

Supplement: S4 Fig — The mVISTA software package was used to determine evolutionary conserved regions among the two sea urchin species indicated, using pmar1b from S. purpuratus as the reference sequence. Each graph show a pairwise alignment with the extent of sequence identity plotted on the Y-axis against the indicated sequence. The grey arrow below each graph shows the extent of sequence used, while filled portions indicate conservation (>70% over 100 bp) of either exons (labeled in blue) or noncoding sequences (pink). Note that significant sequence similarity is found exclusively in the protein coding regions across the two species. (TIF) [file pone.0174404.s005.tif]

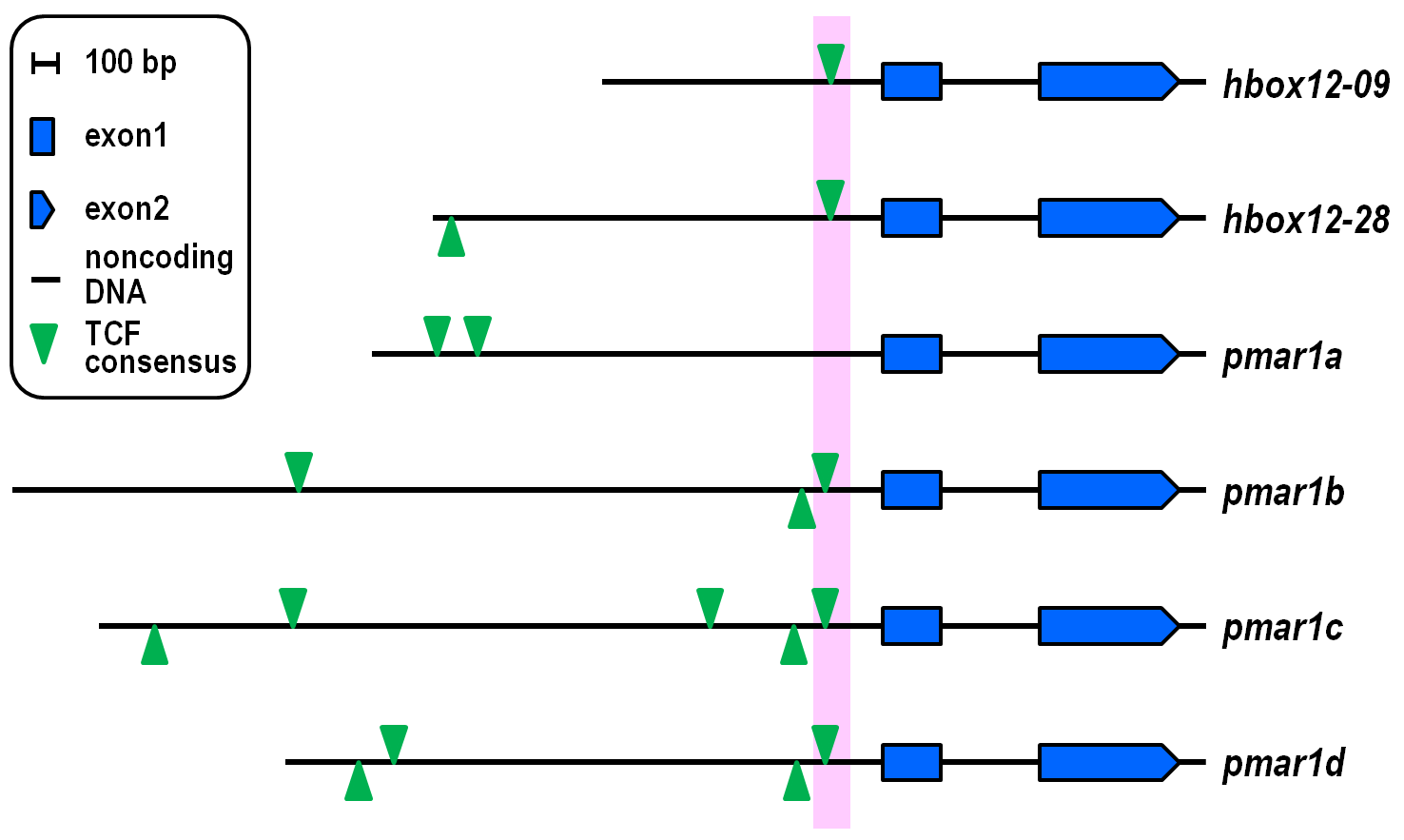

Supplement: S5 Fig — The motifs mapping on the sense and antisense DNA strand are represented respectively above and below the diagram. Pink shading indicates conservation in the relative position of a TCF/LEF motif in 5 out of 6 genes. (TIF) [file pone.0174404.s006.tif]
